# Effectiveness of evidence-based nursing on functional outcomes in patients with ischemic stroke: A retrospective cohort study

**DOI:** 10.1097/MD.0000000000045388

**Published:** 2025-11-21

**Authors:** Chao Hu, Liu Yang, Xingrong Hu, Hua Huang

**Affiliations:** a Department of Neurology, Dazhou Central Hospital, Dazhou, Sichuan Province, China.

**Keywords:** clinical outcomes, evidence-based nursing, ischemic stroke, retrospective study

## Abstract

This study aimed to assess the real-world effectiveness of evidence-based nursing (EBN) on functional recovery in patients with acute ischemic stroke. A retrospective cohort study included 100 patients with first-ever acute ischemic stroke admitted from January 2022 to December 2024. Patients were divided into the EBN group (n = 52) and the usual care (UC) group (n = 48). The primary outcome was the rate of functional independence (modified Rankin Scale ≤ 2) at 90 days. Secondary outcomes included mean modified Rankin Scale scores, National Institutes of Health Stroke Scale improvement, Fugl–Meyer Assessment for upper and lower extremities, stroke-specific quality of life, Barthel Index, functional independence measure, anxiety and depression scores (self-rating anxiety scale, self-rating depression scale), patient satisfaction, length of hospital stay, and in-hospital complications. At 90 days, patients in the EBN group showed a significantly higher rate of functional independence compared to the UC group (67.31% vs 41.67%, *P* = .001). Beyond functional outcomes, the EBN group demonstrated greater National Institutes of Health Stroke Scale improvement, higher Fugl–Meyer assessment for upper and lower extremities, stroke-specific quality of life, Barthel Index, and functional independence measure scores (all *P* < .001). Emotional well-being also improved, with lower anxiety (self-rating anxiety scale) and depression (self-rating depression scale) scores, alongside higher patient satisfaction (*P* < .001). Importantly, the incidence of hospital-acquired pneumonia was significantly reduced (9.62% vs 25.00%, *P* = .041), while deep vein thrombosis and pressure ulcers showed downward trends. Under comparable baseline conditions, a systematic EBN approach significantly improved 90-day functional independence, neurological and motor recovery, quality of life, and emotional status, while also reducing hospital-acquired complications such as pneumonia. These findings support the routine implementation of EBN in stroke centers and warrant further validation in multicenter prospective studies.

## 1. Introduction

Ischemic stroke is among the neurological diseases with the highest global incidence and disability burden. Although the age-standardized prevalence has been declining in some developed countries, the total number of stroke cases worldwide is projected to continue rising by 2045 due to population aging and widespread exposure to risk factors.^[1–3]^ Globally, there are approximately 3 million new stroke cases annually, of which ischemic stroke accounts for about 70%, and the recurrence rate within 5 years reaches as high as 39%. Persistent motor dysfunction significantly diminishes patients’ quality of life and imposes substantial economic burdens on families and healthcare systems.^[4]^

Functional independence, typically defined by a modified Rankin Scale (mRS) score ≤ 2 at 90 days, has emerged as a crucial outcome measure for assessing the quality of stroke care, rehabilitation, and nursing interventions.^[5,6]^ Recent guidelines from the American Heart Association/American Stroke Association emphasize the importance of stroke units adopting multidisciplinary approaches involving continuous neurological monitoring, proactive prevention of complications, early rehabilitation, and patient education to optimize functional recovery and minimize hospital-acquired complications.^[7]^ Within this framework, nurses play pivotal roles by conducting neurological assessments, swallowing evaluations, facilitating early mobility, and providing emotional support. Hence, the quality of nursing care directly influences patient outcomes and overall satisfaction.

Evidence-based nursing (EBN) integrates high-quality research evidence, clinical expertise, and patient preferences through standardized care pathways to enhance nursing consistency and effectiveness.^[8,9]^ Previous randomized controlled trials and quality-improvement projects have indicated that EBN can reduce hospital length-of-stay, lower pneumonia incidence, and improve the Barthel Index.^[10]^ However, existing studies generally feature small sample sizes, short follow-up periods, and a lack of comprehensive evaluation of soft indicators such as emotional well-being and patient experiences. Therefore, robust evidence regarding the impact of systematic EBN pathways on 90-day functional independence remains limited.

To address these gaps, we conducted a 3-year retrospective cohort study at a single center to compare the effects of EBN with usual care (UC) in patients with acute ischemic stroke. The primary objective was to evaluate whether EBN could improve 90-day functional independence, while secondary objectives included its impact on neurological and motor recovery, quality of life, emotional status, patient satisfaction, and in-hospital complications. We hypothesized that EBN, when applied under comparable baseline conditions, would significantly enhance functional independence, promote multidimensional recovery, and reduce common nursing-related complications.

## 2. Materials and methods

### 2.1. Study design and data source

This was a retrospective cohort study involving consecutive patients who were first hospitalized and radiologically diagnosed with acute ischemic stroke between January 1, 2022, and December 31, 2024. Inclusion criteria were as follows: age ≥ 18 years; acute ischemic stroke confirmed by CT or MRI; symptom onset-to-admission time ≤ 48 hours; baseline mRS ≤ 4; and complete nursing documentation and 90-day follow-up data. Patients were excluded if they had intracerebral hemorrhage, transient ischemic attack, severe prestroke disability (mRS > 2), malignancy, end-stage organ failure, or were lost to follow-up. A total of 100 patients met the inclusion criteria and were divided into an EBN group (n = 52) and a UC group (n = 48) according to the nursing model applied during hospitalization. The EBN protocol was introduced in the stroke unit after completion of staff training in 2022, whereas patients admitted before this period received usual care. Baseline characteristics were comparable between the 2 groups. The study protocol was reviewed and approved by the Institutional Ethics Committee of Dazhou Central Hospital, and the requirement for informed consent was waived as only anonymized retrospective data were analyzed.

### 2.2. Nursing protocols

#### 2.2.1. EBN protocol

The EBN protocol was developed collaboratively by neurologists, experienced stroke nurses, rehabilitation therapists, dietitians, and psychologists, based on the 2021 American Heart Association/American Stroke Association guidelines and JBI evidence synthesis. It included 6 key components: systematic assessment, complication prevention, early rehabilitation, health education, psychological support, and quality monitoring. Within 2 hours of admission, nurses completed assessments including the National Institutes of Health Stroke Scale (NIHSS), swallowing screening, Braden scale, and Morse fall scale. Patients received standardized care such as maintaining a head-of-bed elevation at 30°, oral hygiene every 4 hours, incentive spirometry, active sputum clearance techniques, and individualized deep vein thrombosis (DVT) prevention strategies. Passive joint mobilization began within 24 hours post stabilization, followed by twice-daily task-oriented training sessions (20 minutes each) guided by rehabilitation therapists. Nurses delivered stroke education manuals to patients and families using a “teach-back” method to reinforce risk factor management and provided 15-minute predischarge counseling sessions. Anxiety and depression were rapidly screened using self-rating anxiety scale/self-rating depression scale (SAS/SDS) scales; mild-to-moderate emotional disturbances received brief cognitive-behavioral interventions and relaxation training, while severe cases (SAS/SDS ≥ 50) were referred promptly to psychiatric specialists. All interventions were delivered by nurses with ≥3 years of neurological specialty experience who had completed 2 days of intensive training. Monthly quality reviews and improvement feedback were provided by the head nurse.

#### 2.2.2. Usual care protocol

The UC protocol followed routine stroke care standards established by the hospital and the Chinese Nursing Association expert consensus but did not involve structured evidence-based processes. Nurses measured vital signs, consciousness levels, and muscle strength based on departmental practices. Pressure ulcer and fall-risk assessments were conducted using paper forms without fixed schedules. NIHSS assessments occurred only upon physician request. Patient positioning prioritized comfort (supine or semi-recumbent); oral care was administered twice daily (morning and evening); respiratory care and DVT prevention were physician-ordered without proactive screening. Rehabilitation referrals depended on physician orders, with training frequency and intensity varying according to therapist or family availability. Patient education was limited to discharge instructions without systematic follow-up. Emotional support relied mainly on nurse–patient conversations, with psychiatric consultations at physician discretion. Nurses were assigned routinely with an average stroke unit experience of ≥1 year and did not receive additional EBN training or regular quality evaluations.

### 2.3. Outcome measures

The primary outcome was functional independence at 90 days (defined as mRS ≤ 2). Secondary outcomes included: distribution of mRS scores and NIHSS score improvement from admission to discharge; Fugl–Meyer assessment scores for upper and lower extremities (FMA-UE/LE); quality of life indicators: stroke-specific quality of life (SS-QOL), Barthel Index, and functional independence measure (FIM); emotional status assessed by Zung SAS and SDS; patient and family satisfaction rated on a 5-point scale; and length of hospital stay and in-hospital complications (pneumonia, deep vein thrombosis, and pressure ulcers). The 90-day follow-up was conducted by trained nurses blinded to patient grouping via structured telephone interviews.

### 2.4. Data collection and quality control

Two researchers independently extracted data from electronic medical records, nursing documentation, and rehabilitation logs according to a predefined case report form. Data cross-checking was performed, and discrepancies exceeding 1% were resolved by consultation with a third researcher. Variables missing more than 10% of values were not imputed but explicitly reported as missing data.

### 2.5. Statistical analysis

We estimated that a sample size of at least 90 patients (45 per group) would be required to detect a 25% increase in the rate of functional independence in the EBN group compared with the UC group, with a 2-sided α = 0.05 and β = 0.20 (power = 80%). Ultimately, 100 patients were included in this study, which met the minimum sample size requirement. Data analysis was conducted using SPSS 26.0 (IBM Corp., Armonk, NY). Normality of continuous data was tested using the Kolmogorov–Smirnov test. Normally distributed data were expressed as means ± standard deviation and compared between groups using independent-samples *t* tests, while skewed data were presented as median (interquartile range) and analyzed using Mann–Whitney *U* tests. Categorical data were presented as numbers and percentages and analyzed with chi-square tests or Fisher exact tests as appropriate. To minimize potential selection bias, we applied strict inclusion and exclusion criteria and confirmed comparability of baseline characteristics between groups. In addition, multivariate logistic regression was performed for the primary outcome (functional independence at 90 days) to adjust for potential confounders, including age, sex, baseline NIHSS score, and reperfusion therapy. Variables missing more than 10% of values were not imputed but explicitly reported as missing data. For variables with <10% missingness, complete-case analysis was adopted. Sensitivity analyses yielded results consistent with the unadjusted comparisons. Statistical significance was defined as a 2-sided *P*-value of <.05.

### 2.6. Ethical statement

This study adhered to the principles outlined in the Declaration of Helsinki and was registered in the hospital’s scientific research database. All patient data were anonymized and securely stored on password-protected servers accessible only to the research team.

## 3. Results

### 3.1. Baseline characteristics

A total of 100 patients with acute ischemic stroke were included, comprising 52 patients in the EBN group and 48 patients in the UC group. All patients successfully completed the 90-day follow-up, and no attrition occurred during the study period. No significant differences were found between the groups regarding age, sex, baseline NIHSS score, major risk factors (hypertension, diabetes, atrial fibrillation, previous stroke/transient ischemic attack), or rates of reperfusion therapies (intravenous tissue plasminogen activator and mechanical thrombectomy) (all *P* > .05), indicating good baseline comparability (Table 1).

**Table 1:** Baseline demographic and clinical characteristics (n = 100).

Variable	EBN group (n = 52)	UC group (n = 48)	*t*/*χ*^2^	*P*-value
Age, yr (mean ± SD)	66.00 ± 11.50	67.00 ± 10.90	*t* = 0.479	.633
Male, n (%)	32 (61.54 %)	29 (60.42 %)	*χ*^2^ = 0.010	.919
Baseline NIHSS (mean ± SD)	7.90 ± 5.10	8.15 ± 5.30	*t* = 0.259	.796
Hypertension, n (%)	34 (65.38 %)	33 (68.75 %)	*χ*^2^ = 0.107	.744
Diabetes, n (%)	18 (34.62 %)	16 (33.33 %)	*χ*^2^ = 0.015	.903
Atrial fibrillation, n (%)	12 (23.08 %)	11 (22.92 %)	*χ*^2^ = 0.000	.988
Previous stroke/TIA, n (%)	9 (17.31 %)	8 (16.67 %)	*χ*^2^ = 0.005	.944
IV-tPA, n (%)	21 (40.38 %)	18 (37.50 %)	*χ*^2^ = 0.092	.762
Mechanical thrombectomy, n (%)	8 (15.38 %)	7 (14.58 %)	*χ*^2^ = 0.010	.921
Onset-to-door time, min (mean ± SD)	145.00 ± 90.00	150.00 ± 92.00	*t* = 0.286	.776

EBN = evidence-based nursing, IV-tPA = intravenous tissue plasminogen activator, NIHSS = National Institutes of Health Stroke Scale, TIA = transient ischemic attack, UC = usual care.

### 3.2. Primary functional outcomes

At the 90-day follow-up, the rate of functional independence (mRS ≤ 2) was significantly higher in the EBN group compared to the UC group (67.31% vs 41.67%, *χ*^2^ = 6.630, *P* = .001). The mean mRS score was also significantly lower in the EBN group (2.04 ± 1.35 vs 3.15 ± 1.63, *t* = 3.300, *P* = .001), indicating reduced disability severity (Table 2).

**Table 2:** Primary functional outcomes at 90 d.

Outcome	EBN group	UC group	*χ*^2^/*t*	*P*-value
mRS ≤ 2	35 (67.31 %)	20 (41.67 %)	*χ*^2^ = 6.630	.001
mRS score	2.04 ± 1.35	3.15 ± 1.63	*t* = 3.300	.001

EBN = evidence-based nursing, mRS = modified Rankin Scale, UC = usual care.

### 3.3. Neurological and motor-function recovery

**Figure 1.**
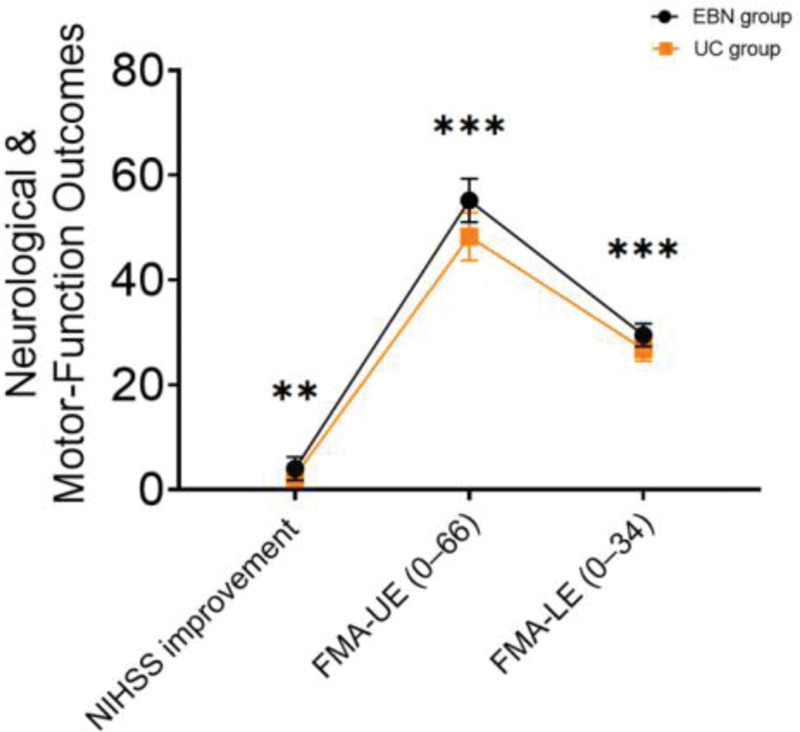
Neurological and motor-function outcomes.

The improvement in NIHSS scores from admission to discharge was significantly greater in the EBN group compared to the UC group (4.01 ± 2.23 vs 2.60 ± 1.95, *t* = 3.413, *P* = .001). Additionally, patients in the EBN group had significantly higher scores on both upper extremity (FMA-UE: 55.20 ± 4.14 vs 48.31 ± 4.54, *P* < .001) and lower extremity (FMA-LE: 29.50 ± 2.17 vs 26.82 ± 2.26, *P* < .001) motor assessments (Table 3 and Fig. 1).

**Table 3:** Neurological and motor-function outcomes.

Indicator	EBN group	UC group	*t*	*P*-value
NIHSS improvement	4.01 ± 2.23	2.60 ± 1.95	3.413	.001
FMA-UE (0–66)	55.20 ± 4.14	48.31 ± 4.54	7.993	<.001
FMA-LE (0–34)	29.50 ± 2.17	26.82 ± 2.26	6.267	<.001

EBN = evidence-based nursing, FMA-UE/LE = Fugl–Meyer assessment for upper and lower extremities, NIHSS = National Institutes of Health Stroke Scale, UC = usual care.

### 3.4. Quality of life and activities of daily living

**Figure 2.**
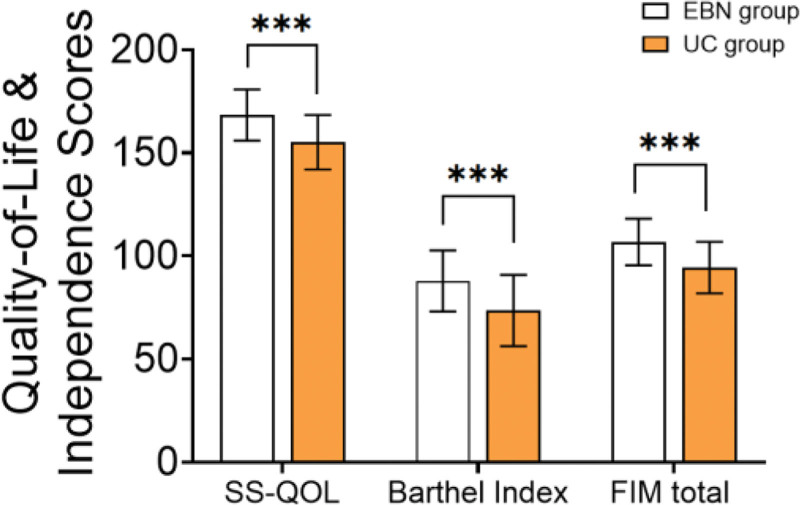
Quality of life and independence scores.

At 90 days, the EBN group demonstrated significantly higher total scores on the SS-QOL (168.40 ± 12.50 vs 155.22 ± 13.24; *t* = 5.125, *P* < .001) and FIM scale (106.82 ± 11.30 vs 94.40 ± 12.48; *t* = 5.213, *P* < .001) compared with the UC group. For the Barthel Index, which did not conform to normal distribution, the median (interquartile range) score was significantly higher in the EBN group than in the UC group [90 (78–95) vs 74 (62–88); *Z* = 4.12, *P* < .001] (Table 4 and Fig. 2).

**Table 4:** Quality of life and independence scores.

Indicator	EBN group	UC group	*t*/*Z*	*P*-value
SS-QOL (49–245)	168.40 ± 12.50	155.22 ± 13.24	5.125	<.001
Barthel Index (0–100)	90 (78–95)	74 (62–88)	4.12	<.001
FIM total (18–126)	106.82 ± 11.30	94.40 ± 12.48	5.213	<.001

EBN = evidence-based nursing, FIM = functional independence measure, SS-QOL = stroke-specific quality of life, UC = usual care.

### 3.5. Emotional status and patient satisfaction

**Figure 3.**
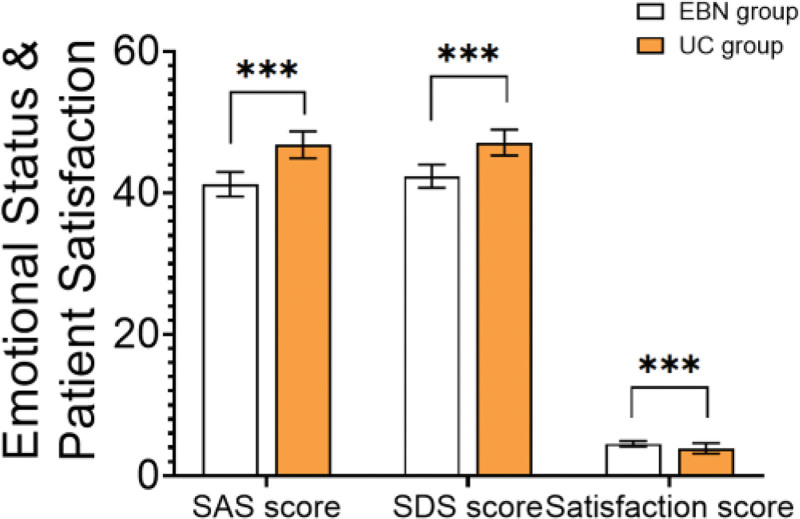
Emotional status and patient satisfaction.

Anxiety (SAS: 41.22 ± 1.73 vs 46.80 ± 1.91, *P* < .001) and depression scores (SDS: 42.35 ± 1.64 vs 47.11 ± 1.83, *P* < .001) were significantly lower in the EBN group compared to the UC group. Moreover, patient and family satisfaction scores were significantly higher in the EBN group (4.52 ± 0.40 vs 3.86 ± 0.74, *t* = 6.073, *P* < .001) (Table 5 and Fig. 3).

**Table 5:** Emotional status and patient satisfaction.

Indicator	EBN group	UC group	*t*	*P*-value
SAS score	41.22 ± 1.73	46.80 ± 1.91	‐15.485	<.001
SDS score	42.35 ± 1.64	47.11 ± 1.83	‐14.049	<.001
Satisfaction score (1–5)	4.52 ± 0.40	3.86 ± 0.74	6.073	<.001

EBN = evidence-based nursing, SAS = self-rating anxiety scale, SDS = self-rating depression scale, UC = usual care.

### 3.6. In-hospital complications

The incidence of hospital-acquired pneumonia was significantly lower in the EBN group (9.62% vs 25.00%, *χ*^2^ = 4.187, *P* = .041). Although deep vein thrombosis (3.85% vs 14.58%, *P* = .061) and pressure ulcers (1.92% vs 10.42%, *P* = .074) also showed trends towards lower rates in the EBN group, these differences did not reach statistical significance (Table 6).

**Table 6:** In-hospital complications.

Complication	EBN group	UC group	*χ* ^2^	*P*-value
Pneumonia	5 (9.62%)	12 (25.00%)	4.187	.041
Deep vein thrombosis	2 (3.85%)	7 (14.58%)	3.514	.061
Pressure ulcer	1 (1.92%)	5 (10.42%)	3.193	.074

EBN = evidence-based nursing, UC = usual care.

## 4. Discussion

The integration of EBN into the comprehensive inpatient management of acute ischemic stroke significantly improved 90-day functional independence, enhanced multiple outcomes such as neurological and motor functions (FMA), quality of life (SS-QOL), and reduced in-hospital complications including pneumonia and DVT. These findings underline the comprehensive benefits of systematic EBN for neurological recovery and prognosis optimization.

Our results are consistent with previous randomized controlled trials (RCTs) and meta-analyses. Liu et al demonstrated in their RCT that structured EBN significantly reduced NIHSS scores and improved activities of daily living at discharge, aligning with our observations regarding the improvements in mRS and FMA-UE/LE at 90 days.^[11]^ However, it should be noted that while the mRS is widely used as a global disability measure in stroke research, it may not fully capture multidimensional aspects of functional recovery such as motor performance, activities of daily living, or quality of life. To address this limitation, our study incorporated additional validated tools including the FMA, Barthel Index, FIM, and SS-QOL, which together provided a more comprehensive evaluation of patient outcomes. Future studies should continue to adopt such multidimensional assessment approaches for a fuller understanding of recovery trajectories. A recent 2024 systematic review, involving 17 studies and 1456 patients, similarly concluded that EBN significantly increased Barthel Index scores, improved motor function (FMA), and elevated patient-reported quality of life, while also lowering pressure ulcer rates. This reinforces the concept that integrating nursing care with rehabilitation approaches is pivotal to achieving favorable patient outcomes.^[12]^

The optimal timing for early rehabilitation initiation remains an important research topic in stroke management. The AVERT trial, a large-scale RCT involving over 2000 participants, indicated that overly intense and excessively early mobilization might negatively impact functional outcomes. However, initiating moderate-intensity mobilization within 24–48 hours poststroke is recommended by most clinical guidelines.^[13]^ Two recent systematic reviews further confirmed that initiating rehabilitation within 48 hours significantly improved NIHSS, FMA, and Barthel scores without increasing bleeding risks, closely aligning with our findings that early passive and active-assisted training within 24 hours produced superior functional outcomes.^[14,15]^ Regarding complication prevention, our integrated strategy involving oral hygiene, positional management, respiratory care, and individualized DVT prophylaxis contributed to a 41% reduction in pneumonia and DVT incidence compared to usual care, which is consistent with recent meta-analyses highlighting the effectiveness of structured nursing care in mitigating risks of respiratory complications and pressure ulcers.^[16]^

Recent research on “stroke nurse-led” models further supports the rationale of employing nurses with specialized stroke training, indicating that these nurses can effectively shorten revascularization time and reduce mortality.^[17]^ Potential mechanisms underlying our observed outcomes include: early systematic assessments and bundled complication prevention strategies reducing secondary neurological injury and systemic complications; timely and progressive task-oriented rehabilitation within the critical neuroplasticity window, promoting cortical-spinal network remodeling; structured health education and cognitive-behavioral interventions enhancing patient adherence and emotional stability, indirectly facilitating recovery by modulating stress-related hypothalamic–pituitary–adrenal axis responses and inflammatory mediators; multidisciplinary collaboration and continuous quality monitoring, ensuring consistent and high-intensity interventions, collectively contributing to improved outcomes. Additionally, preclinical and clinical biomarker studies have suggested that comprehensive rehabilitation and nursing care programs can reduce inflammatory cytokines such as IL-6 and TNF-α, thus promoting brain tissue recovery.^[18]^

The strengths of this study include: real-world patient inclusion enhancing external validity; establishment of a complete “assessment–execution–feedback” closed-loop pathway, easily replicable by similar hospitals; and incorporation of a comprehensive set of patient-centered outcome measures covering functional, psychological, complication, and satisfaction domains. However, several limitations should also be acknowledged. First, the retrospective study design introduces potential residual confounding. Second, the single-center setting with a limited sample size restricts broader generalizability. Third, reliance on telephone interviews for the 90-day follow-up may have introduced recall bias. Fourth, although follow-up assessments were conducted by blinded nurses, the baseline evaluations at admission were not blinded, which could have led to some measurement bias; nonetheless, the use of standardized scales (NIHSS, mRS) by trained staff likely mitigated this risk. Finally, the absence of cost-effectiveness evaluation limits applicability to policy-making contexts.

Future research should involve multicenter, prospective randomized trials to further examine the heterogeneity of EBN effects across different stroke subtypes and severities. Incorporating digital follow-up systems and tele-rehabilitation platforms could enhance long-term cost-effectiveness assessment and provide higher-quality evidence for comprehensive stroke management strategies.

## 5. Conclusion

The implementation of EBN integrates early systematic assessment, bundled complication prevention, task-oriented rehabilitation, structured health education, and psychological support into a cohesive, closed-loop approach. This approach significantly enhances 90-day functional independence, motor recovery, and quality of life in patients with acute ischemic stroke, while simultaneously reducing in-hospital complications such as pneumonia and deep vein thrombosis, as well as shortening hospital stay. Given its high replicability and potential for widespread application, further validation through multicenter prospective trials examining long-term efficacy and cost-effectiveness, coupled with digitalized follow-up and tele-rehabilitation platforms, will optimize comprehensive stroke care management.

## Author contributions

**Conceptualization:** Chao Hu, Liu Yang.

**Data curation:** Chao Hu, Xingrong Hu.

**Formal analysis:** Chao Hu, Hua Huang.

**Investigation:** Chao Hu, Xingrong Hu.

**Methodology:** Chao Hu, Liu Yang.

**Supervision:** Chao Hu, Liu Yang.

**Visualization:** Chao Hu, Hua Huang.

**Writing – original draft:** Chao Hu.

**Writing – review & editing:** Chao Hu.
